# Understanding the impact of *in vitro* transcription byproducts and contaminants

**DOI:** 10.3389/fmolb.2024.1426129

**Published:** 2024-07-10

**Authors:** Robin Lenk, Werner Kleindienst, Gábor Tamás Szabó, Markus Baiersdörfer, Gábor Boros, Jason M. Keller, Azita J. Mahiny, Irena Vlatkovic

**Affiliations:** BioNTech SE, Mainz, Germany

**Keywords:** *in vitro* transcription reaction (IVT), double-stranded RNA (dsRNA), abortive transcripts, IVT byproducts, IVT contaminants, mRNA purification

## Abstract

The success of messenger (m)RNA-based vaccines against SARS-CoV-2 during the COVID-19 pandemic has led to rapid growth and innovation in the field of mRNA-based therapeutics. However, mRNA production, whether in small amounts for research or large-scale GMP-grade for biopharmaceutics, is still based on the *In Vitro* Transcription (IVT) reaction developed in the early 1980s. The IVT reaction exploits phage RNA polymerase to catalyze the formation of an engineered mRNA that depends on a linearized DNA template, nucleotide building blocks, as well as pH, temperature, and reaction time. But depending on the IVT conditions and subsequent purification steps, diverse byproducts such as dsRNA, abortive RNAs and RNA:DNA hybrids might form. Unwanted byproducts, if not removed, could be formulated together with the full-length mRNA and cause an immune response in cells by activating host pattern recognition receptors. In this review, we summarize the potential types of IVT byproducts, their known biological activity, and how they can impact the efficacy and safety of mRNA therapeutics. In addition, we briefly overview non-nucleotide-based contaminants such as RNases, endotoxin and metal ions that, when present in the IVT reaction, can also influence the activity of mRNA-based drugs. We further discuss current approaches aimed at adjusting the IVT reaction conditions or improving mRNA purification to achieve optimal performance for medical applications.

## Introduction

Following the success of the first clinically approved mRNA-based vaccines during the COVID-19 pandemic, mRNA therapeutics hold tangible promise as safe and effective biologics of the future, expected to prevent, improve, or cure a variety of diseases with unmet medical need. The active substance and basis of this new class of drugs is a designed, synthetic single-stranded messenger RNA (mRNA) molecule. In mammalian cells, endogenous mRNA is normally transcribed from DNA in the nucleus by RNA polymerase II, in the form of a pre-mRNA transcript, then it is 5′ capped, 3′ polyadenylated, spliced to remove introns, and finally transported to the cytoplasm where it can be translated into a specific protein. The synthetic mRNA used for therapeutics is instead produced enzymatically in solution, purified, packaged into a formulated lipid carrier, and delivered to the cytoplasm by way of endocytosis. To artificially generate mRNA in solution under cell-free conditions, the technique of *in vitro* transcription (IVT) was developed and utilizes a phage RNA polymerase that can recognize diverse types of DNA templates. However, due to the properties and components of the IVT reaction, distinct nucleotide-based byproducts may be formed in addition to the full-length mRNA molecule templated by the DNA. Moreover, depending on the starting materials or equipment used at different stages of the production and purification processes, other types of process related, non-nucleotide-based contaminants may be introduced. Any of the product or process related contaminants that reach the formulated drug can potentially activate pattern recognition receptors (PRRs), causing a host immune response that may negatively impact mRNA-encoded protein expression. Thus, several aspects of mRNA production are necessary to implement, and they are under continuous development: (1) optimizing the IVT reaction components and conditions; (2) purification procedures; and (3) stringent quality control using analytical methods that can detect and characterize byproducts and contaminants. Previous reviews and assessment reports have already discussed in detail the specific analytical methods and quality controls currently used for mRNA-based drug manufacturing (European Medicines Agency, 2021; Schoenmaker et al., 2021; Daniel et al., 2022; European Medicines Agency, 2022; BioPhorum Operations Group Ltd, 2023). In this review, we will highlight the various types of IVT reaction byproducts and contaminants as well as their mechanisms of action that may influence the quality and performance of mRNA-based therapeutics.

## *In vitro* transcription reaction

A synthetic mRNA is typically produced in a cell-free enzymatic IVT reaction consisting of a DNA template to guide the sequence, free nucleoside triphosphate (NTP) monomers, a DNA-dependent RNA polymerase that transcribes the mRNA directly from the template, and an optimized buffer. The quality of the components and their stoichiometric ratios in the reaction mixture, physical parameters of the process such as duration, pH or temperature, and the design of the mRNA encoded on the template can highly influence the yield and quality of the final product (Triana-Alonso et al., 1995; Mu et al., 2018). The design of the encoded mRNA, particularly its length, the sequence of the untranslated regions (UTR) and open reading frame, the number of repetitive sequences and homogenic parts, and the templated poly(A) tail, can directly influence the performance of the IVT process (Conrad et al., 2020; Leppek et al., 2022). Most commonly, circular plasmid DNA (pDNA) containing the specific RNA polymerase promoter sequence is linearized and used as the template for IVT reactions. How the plasmid is linearized, including the selected type and concentration of restriction enzymes, the buffer used, duration of the reaction, and the downstream purification process, can influence the quality of the DNA template and consequently the final mRNA product. A chemically synthesized ‘doggybone’ DNA (dbDNA™) vector, which is a linear, closed-end molecule produced entirely in a cell-free process, can also be used (Touchlight Genetics Ltd, 2024). Similarly, synthetic linear DNA with either two open 5′ and 3′ ends (oeDNA™) or one open and one hairpin end (opDNA™) are available whereby the open ends can be chosen to include a blunt end or an overhang (4basebio PLC, 2024). Such synthetic DNA templates for IVT reactions, however, are still in development. Alternatively, PCR amplified DNA can serve as an effective template for the IVT reaction (Brunelle and Green, 2013). As necessary building blocks of mRNA, the IVT reaction contains adenine (A), guanine (G), cytosine (C) and uridine (U), and/or their isomers or modified derivatives. The choice of nucleotides and their starting ratios, or changing their ratios during IVT respectively (“feeding” strategy), can influence the transcription efficiency and the amount and types of byproducts produced during the reaction (Skok et al., 2022; Ziegenhals et al., 2023). Currently, the most widely used RNA polymerase is the bacteriophage T7 RNA polymerase. Selection of the RNA polymerase might significantly influence the IVT reaction. Ideally, the RNA polymerase should have a high transcriptional yield and fidelity, full activity under variable reaction conditions, and the ability to incorporate modified NTPs with high fidelity. 5′ cap analogs can be added to the reaction mixture for co-transcriptional capping, which reduces the need for later purification steps and also increases the yield of full-length mRNA (Ramanathan et al., 2016; Henderson et al., 2021). If co-transcriptional capping is not performed, a 5′ cap can be enzymatically added post-transcriptionally using RNA triphosphatase, guanylyl-transferase and guanine methyltransferase to first form Cap0 and then with the subsequent addition of mRNA Cap 2′-O-Methyltransferase to form Cap1 (Ensinger et al., 1975). Similarly, while a poly(A) tail is often directly encoded by the template, it can also be added or extended post transcriptionally using Poly(A) polymerase. mRNA yields and quality, including the amounts of byproducts, are significantly influenced by the stoichiometric ratios of the ingredients, the physical parameters of the reaction such as pH and temperature, and the duration of the reaction. To select optimal conditions, the sequence and the length of the mRNA and the selected RNA polymerase must be considered. With optimization of the IVT reaction, a dramatic reduction of unwanted byproducts can be achieved. Moreover, non-obligatory ingredients can be added to enhance yield and quality or reduce downstream purification steps. Such auxiliary reagents can be used to enhance transcription (e.g., spermidine, DMSO, urea) (Moruzzi et al., 1975; Chen and Zhang, 2005; Francis et al., 2024), to support polymerase enzymatic activity (e.g., dithiothreitol) (Kartje et al., 2021) or to reduce mRNA degradation (e.g., RNase inhibitors) and byproduct formation (e.g., denaturing agents; pyrophosphatase) (Cunningham and Ofengand, 1990; Piao et al., 2022).

## IVT byproducts and their biological impacts

**FIGURE 1:**
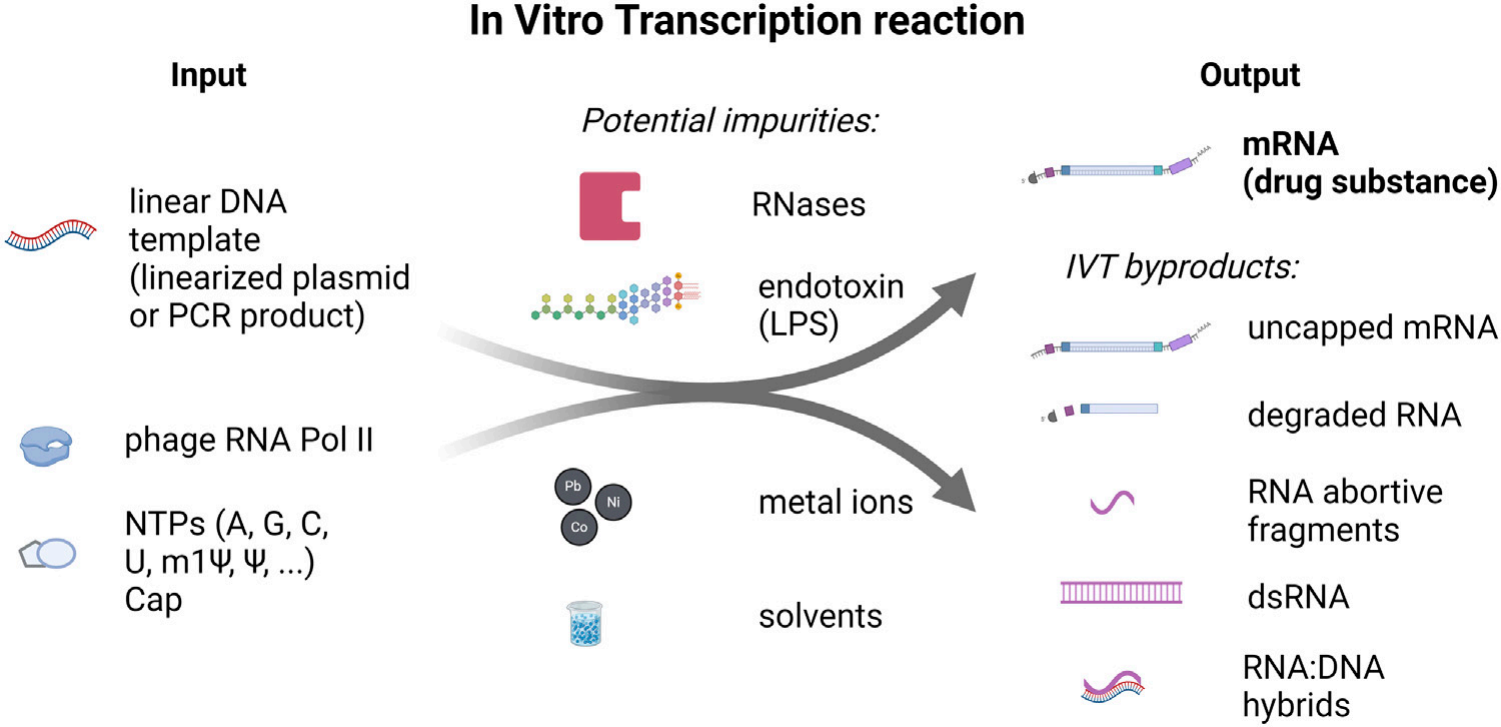
*In vitro* transcription reaction input and output components and potential impurities. On the left side, linear DNA template (linearized plasmid and PCR product) and nucleoside-triphosphates (NTPs), including N1-Methylpseudouridine-5′-Triphosphate (m1Ψ), and RNA Polymerase (RNA Pol II) are listed as inputs to the IVT reaction. On the right side, outputs of the IVT reaction: mRNA (drug substance) and IVT byproducts are shown. In addition, potential impurities that can be introduced to the reaction through the raw materials or during the production process are listed in the middle.

**FIGURE 2:**
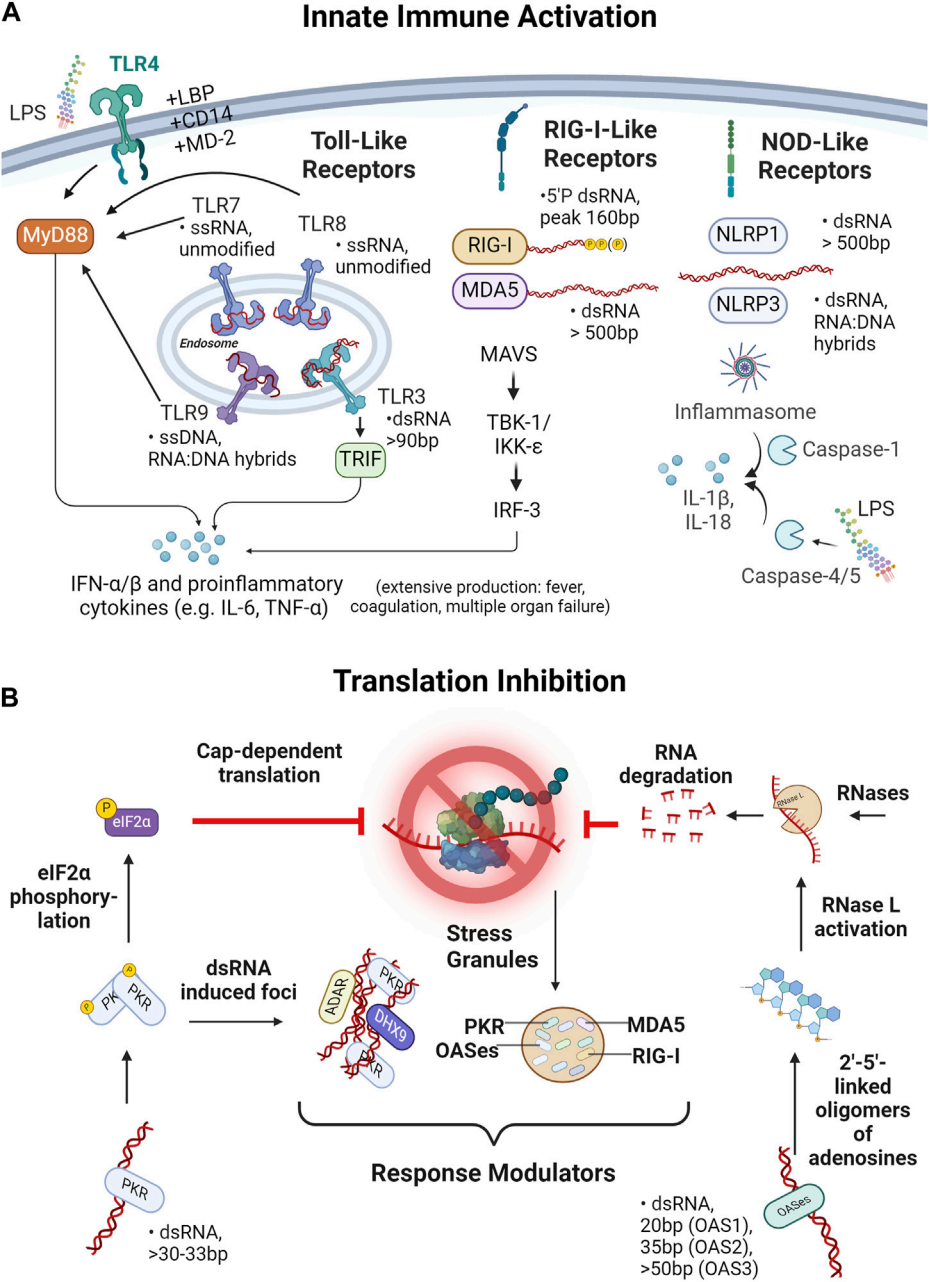
IVT reaction byproducts and contaminants and their impact in transfected cells. **(A)** Impact of IVT reaction byproducts and contaminants on innate immune activation is shown. Depicted are three receptor families, namely, Toll-like receptors (TLRs), retinoic acid inducible gene I-like receptors (RIG-I-like receptors) and NOD-like receptors (NLRs) known to detect ssRNA, dsRNA and RNA:DNA hybrids of various lengths. Activation of TLR4, 7, 8 or 9 leads via the MyD88-pathway and TLR3 via TRIF to the secretion of IFN- α/β and proinflammatory cytokines such as IL-6 or IFN-γ. Likewise, the RIG-I-like receptors, MDA5 and RIG-I lead via MAVS, TBK-1/IKK-ε and IRF-3 to a secretion of proinflammatory cytokines. In contrast, the NOD-like receptors NLRP1 and NLRP3 trigger inflammasome assembly and via caspase-1 lead to IL-1β and IL-18 release. Apart from the immunostimulatory activity of IVT byproducts of “nucleic nature”, detection of bacterial lipopolysaccharide (LPS) via TLR4 or caspase-4/5 and subsequent inflammasome activation triggers innate immune activation. **(B)** Mechanisms of translational inhibition due to effects of dsRNA byproducts are depicted. dsRNA can be recognized by protein kinase R (PKR) or 2′–5′ Oligoadenylate synthetases (OASes). Two PKR-molecules can autophosphorylate upon dsRNA binding, allowing phosphorylation of eIF2 transcription initiation factor. eIF2 plays a central role in initiating translation, thus, phosphorylation of eIF2 inhibits cap-dependent translation of RNA. OASes, on the other hand, act through linkage and oligomerization of ATP leading to activation of RNase L and, in turn, to the degradation of ssRNA (i.e., the drug product). Both PKR and OASes, as well as the RIG-I,-like receptors MDA5 and RIG-I participate in the formation of dsRNA-induced foci, so called stress granules which are thought to amplify or control dsRNA sensing responses.

The major forms of IVT byproducts are short or long ssRNA, dsRNA or RNA:DNA hybrids (Figure 1). Since many viruses rely on RNA to carry their genetic information, the uptake of such material has been determined through evolution to be a potential danger signal for the cell. The nucleotide-based byproducts are sensed by a multitude of immune pathways leading to inflammation, growth inhibition or even cell death (Figure 2) (Sahin et al., 2014; Devoldere et al., 2016; Vlatkovic, 2021). Even the purest single-stranded, uridine containing mRNA products show a certain degree of immunogenicity mediated through Toll-like receptor (TLR) 7 and 8 (Heil et al., 2004). However, by incorporating modified nucleotides and removing the byproducts from the final mRNA transcription reaction, it is possible to create a therapeutic product that is nearly immune silent (Karikó et al., 2005; Karikó et al., 2008; Karikó et al., 2011; Andries et al., 2015). For certain applications where relatively high doses under repetitive intravenous administration would be required without causing an unwanted immune response, mRNA-based therapeutics are being further improved (Vlatkovic, 2021). Some critical elements serving as the basis for such improvements are a better understanding of the types of byproducts that can be produced in the IVT reaction, tactics to control their quantities, and clarifying their potential biological impacts. Below we summarize progress that has been made in this regard.

### Abortive transcripts

The first evidence that RNA polymerases can produce short, abortive transcripts derived from the transcription start site was given by Bellamy et al. (1972) when they sequenced short RNA oligonucleotides in the virions of reoviruses. Later, a steady-state release of di-nucleotide RNA oligos in the presence of only two species of RNA nucleotides (ATP and UTP) was described using λb2 DNA and *Escherichia coli* RNA polymerase (Johnston and McClure, 1976). More importantly, the authors also showed that in the presence of all four RNA nucleotides, ≤10% of the total RNA synthesized resembled abortive products (i.e., RNA dinucleotides).

As stated above, T7 RNA polymerase is commonly used for industrial production of RNA therapeutics because of its high fidelity and processivity. In 1987, the first RNA using T7 RNA polymerase and synthetic DNA templates was synthesized (Milligan et al., 1987). As reported previously for *E. coli* RNA polymerase (Johnston and McClure, 1976; McClure et al., 1978), Milligan et al. found that T7 RNA polymerase also produces numerous shorter sequences resembling the 5′-end of the template strand. The amounts and specific sequences of abortive products varied but depended on the +1 to +6 sequence of the synthetic DNA template (Milligan et al., 1987).

To date, many studies have contributed to our understanding of the complex mechanism by which T7 RNA polymerase initiates RNA transcription. Briefly, after the T7 RNA polymerase binds its promoter (Rosa, 1979), bending the DNA strand and thereby opening it (Ujvári and Martin, 2000; Tang and Patel, 2006a; 2006b; Tang et al., 2008), the enzyme starts synthesizing the first nucleotides of the RNA strand. The growing RNA:DNA hybrid pushes against the promoter-bound region of the T7 RNA polymerase and causes a 40° rotation after 3 to 7 nt have been synthesized. (Brieba and Sousa, 2001; Mukherjee et al., 2002; Tahirov et al., 2002; Ma et al., 2005; Bandwar et al., 2007; Durniak et al., 2008). Only after the synthesis of 9 to 12 nt does a drastic 220° rotation occur, which releases the upstream promoter interactions and thus enables the transition of the T7 RNA polymerase into a stable elongation complex (EC) for full-length RNA synthesis (Brieba and Sousa, 2001; Mukherjee et al., 2002; Tahirov et al., 2002; Ma et al., 2005; Bandwar et al., 2007; Durniak et al., 2008; Tang et al., 2009; Ramírez-Tapia and Martin, 2012). During the entire initiation process, abortive byproducts with a length of 2 to 13 nt can be released (Martin et al., 1988; Brieba and Sousa, 2001; Ikeda and Richardson, 1986; Jia and Patel, 1997; Tang et al., 2008).

Koh et al. (2018) were able to resolve the transcription initiation process at near base-pair resolution enabling a thorough understanding of abortive cycling and processive RNA synthesis, respectively. They found two distinct pathways that can lead to the release of abortive products. Either the T7 RNA polymerase remains bound to the DNA and re-starts RNA synthesis after a release of abortive products (‘RNA polymerase recycling’) or, secondly, T7 RNA polymerase dissociates with or after RNA release and the next round of RNA synthesis starts only after binding of a new T7 RNA polymerase molecule (‘RNA polymerase exchange’). Moreover, in the same study, the authors were able to develop a probabilistic model for the three possible outcomes of T7 RNA polymerase mediated transcription (on DNA templates bearing a class III consensus promoter) that are: elongation, dissociation, and abortive initiation (Koh et al., 2018). Interestingly, they could show that the majority (about 56%) of total initiation events with T7 RNA polymerase molecules result in elongation (i.e., full-length RNA synthesis) without generating abortive byproducts. The remaining initiation events lead to the generation of abortive byproducts whereby the T7 RNA polymerase molecules can either still produce the full-length product after ‘re-starting’ the transcription or completely fail to undergo the transition into the elongation complex. Those molecules that completely fail to transition into the elongation phase account for about six-times as many abortive byproducts as those molecules that can still proceed with full-length RNA synthesis. This underscores that failed transitions into the elongation complex not only reduce the productive capacity of RNA polymerase but are also a major contributor to byproduct formation (Koh et al., 2018).

Of note, abortive initiation can be regarded as a universal feature of RNA polymerases (Alhadid et al., 2017) occurring in prokaryotic (Hsu, 2002), viral (e.g., T7 phage as described, reovirus (Bellamy et al., 1972; Farsetta et al., 2000) or brome mosaic virus (Sun et al., 1996)) and eukaryotic RNA polymerases (e.g., shown for yeast by Bhargava and Kassavetis, 1999). Recently, abortive transcripts were also shown to occur in (yeast) mitochondria (Goovaerts et al., 2023) and in mouse serum and liver (Qin et al., 2024). From an evolutionary perspective, abortive RNA byproducts might have served as the first primers for DNA replication (Matsumoto, 1994). Interestingly, abortive products can exert anti-termination activity by interfering with RNA terminator hairpin formation (Lee et al., 2010) and thus regulate the activation of the late genes of viral replication after accumulating during transcription of earlier genes. Moreover, very short RNAs (2–4 nt in length, termed nanoRNAs) are thought to prime transcription in bacteria, thereby regulating gene expression (Goldman et al., 2011; Nickels and Dove, 2011; Vvedenskaya et al., 2012). Assuming abortive byproducts reach the cytosol of transfected cells, there might occur yet to be defined interactions with intrinsic RNAs or PRRs underlining the need for further research on IVT byproducts.

### Double-stranded RNA (dsRNA)

Even though RNA itself inherently has a certain immunogenicity, the immunogenicity associated with IVT products can be induced by dsRNA (Karikó et al., 2011) (Figure 2A). Two main pathways for how dsRNA byproducts arise during IVT have been identified. One is by way of a promoter-independent, DNA-dependent transcription of the nontemplate strand (Mu et al., 2018) and the other is by RNA-dependent 3′ end additions (Cazenave and Uhlenbeck, 1994; Triana-Alonso et al., 1995). With regard to 3′ end additions, it was shown by Gholamalipour et al. that most 3′ additions arise via a *cis* mechanism, whereby the end of the RNA folds back onto itself and the T7 RNA polymerase rebinds to this dsRNA hairpin and starts extending it with the nontemplate sequence (Gholamalipour et al., 2018). This rebinding and extension of the RNA by the T7 RNA polymerase is independent of the DNA template and can even be observed on chemically produced run-off RNAs (Gholamalipour et al., 2018). Double-stranded RNA can also form when the T7 RNA polymerase switches strands at the end of the DNA template and thereby transcribes a complimentary mRNA strand. This mechanism was shown, although promoter-independent, to have a certain sequence specificity for the 3′ end of the DNA template (Sequence: GTG​GAA​TAC​CCA​TTC​GAC​ATT​CTC​CC) even though the mechanism underlying this effect has not been investigated yet (Mu et al., 2018; Wu et al., 2020). In both cases, the 3′ extension as well as the promoter-independent transcription initiation, the exact mechanism of how the T7 RNA polymerase impacts the formation of dsRNA byproducts remains elusive. In general, upon binding to the T7 promoter, the T7 RNA polymerase forms an initiation complex (IC) that transitions into the more stable elongation complex (EC) after transcription of ∼10 nucleotides (Cheetham and Steitz, 1999). It has been hypothesized that the dsRNA byproducts mentioned above are driven by the EC of the T7 RNA polymerase, which is either directly binding to the nontemplate strand or to the run-off product that folds into a hairpin and starts producing the undesired nontemplate RNA strand. This suggests that stabilizing the IC or decreasing the IC to EC transition barrier might decrease the formation of dsRNA (Dousis et al., 2023) but further investigation is needed.

Once inside a cell, dsRNA can be detected by several sensor proteins that lead to different response cascades. One class of well-known dsRNA sensors are the cytosolic RIG-I (retinoic acid inducible gene I)-like receptors (RLRs): RIG-I, melanoma differentiation-associated protein 5 (MDA5) and LGP2 (Rehwinkel and Gack, 2020) (Table 1; Figure 2A). While RIG-I and MDA5 are direct activators of pathways leading to the release of type I interferons, LGP2 is hypothesized to be a regulator of RIG-I and MDA5 (Satoh et al., 2010; Kato et al., 2011; Childs et al., 2013). RIG-I and MDA5 both activate the MAVS pathway upon filamentation and the subsequent ubiquitination and oligomerization of their two N-terminal CARD domains (Peisley et al., 2012; Peisley et al., 2013). MAVS recruits TRAF3/6 which subsequently activates the kinase complexes TBK1 and IKK, which in turn leads to activation of IRF3/7 and NF-κB, respectively (Wu et al., 2023). While the downstream signaling cascades of the two receptors are very similar, the dsRNA species that they each recognize are distinct. MDA5 forms a filament by binding multiple monomers to one dsRNA. The downstream signal strength increases with the length of the dsRNA duplex but requires a minimum length of ∼500–1000bp to initiate and is sequence independent (Peisley et al., 2011; Peisley et al., 2012). Since most RNAs produced with IVT for clinical applications are longer than 500bp and can even be significantly longer, dsRNA byproducts of these RNAs would have the potential to trigger MDA5. RIG-I, on the other hand, is triggered by dsRNA species with a di- or triphosphate on the 5′end, and where the 2′-O position of the terminal nucleotide is unmethylated (Hornung et al., 2006; Schmidt et al., 2009; Goubau et al., 2014; Schuberth-Wagner et al., 2015). Length-dependent activation of RIG-I seems to follow a bell-shaped curve with an apparent peak at ∼160bp (Cadena et al., 2019). The IVT byproducts that would fit this size profile and activate RIG-I are uncapped dsRNAs. However, it is possible that when T7 RNA polymerase switches strands, it effectively leaves a triphosphate at the 5′ end of the nontemplated RNA strand, even on an initially capped transcript, and this may also activate RIG-I. While RNA modifications like m6-adenosine (m6A), pseudouridine (Ψ) and 1-methylpseudouridine (m1Ψ) have been shown to strongly reduce the downstream activity of RIG-I, the same effect has not been observed for MDA5 (Table 1) (Peisley et al., 2013; Durbin et al., 2016; Mu et al., 2018).

**TABLE 1:** Impacts of IVT reaction byproducts on immune signaling sensors and dsRNA-dependent enzymes in the cell.

	Family name	Name	Recognition species	Evading RNA modifications
Immune Signaling Sensors	RLR	RIG-I	di-, triphosphate 5′end, peak activity 160bp	Ψ, m1 Ψ, m6A
		MDA5	>500bp	none
	NLR	NLPR1	ND	ND
		NLRP3	>500bp	ND
	TLR	TLR3	>90bp	m6a, s2u
dsRNA-dependent Enzymes	PKR	PKR	>30 – 33bp	Ψ
	OAS	OAS1	∼20bp	Ψ, m6a, s2u
		OAS2	∼35bp	
		OAS3	>50bp	

Abbreviations: RLR, RIG-I (retinoic acid inducible gene I)-like receptors; MDA5, melanoma differentiation-associated protein 5; NLR, NOD-like receptors; TLR, Toll-like receptor; PKR, protein kinase R; OAS, 2′-5′-oligoadenylate synthetase; ND, not determined.

Another class of sensors capable of detecting dsRNA are the NOD-like receptors (NLRs) (Table 1; Figure 2A). While there are about 20 known representatives of this type in humans, only NLRP1 and NLRP3 have been shown to be activated by RNA duplexes (Allen et al., 2009; Bauernfried et al., 2021). These two receptors both have a pyrin domain (PYD), through which they interact with apoptosis-associated speck-like protein containing a CARD (ASC) and form the inflammasome to activate caspase1. Caspase1 leads to the activation of the proinflammatory cytokines IL-1β and IL-18 (Kienes et al., 2021; Ohto, 2022; Xu and Núñez, 2023). For NLRP1 it has been shown that a dsRNA duplex length >500bp is needed for activation, while for NLRP3 the precise nature of the stimulatory dsRNA species remains to be identified (Table 1) (Allen et al., 2009; Bauernfried et al., 2021). Thus, NLRP1 appears to sense the same species of byproducts as MDA5. If and how RNA modifications influence recognition by NLR has not been investigated yet.

One of the oldest classes of PRRs in evolution are the Toll-like receptors (TLRs). In humans the TLR family comprises 10 types (TLR1-10), with only TLR3 able to sense dsRNA. In contrast to the RLRs and NLRs, TLR3 is present in the endosomal membrane and senses dsRNA inside the endosome (Figure 2A). In fact, the low pH of the early to late endosomes is crucial for dsRNA binding to TLR3 (Leonard et al., 2008). Upon binding dsRNA, TLR3 homodimerizes, forms clusters on longer dsRNA and recruits TIR-domain-containing adapter-inducing interferon (TRIF). Downstream of TRIF, TRAF3 induces activation of TBK1, again leading to the transcription of type I interferons and proinflammatory cytokines by IRF3/7 and NF-κB (Fitzgerald and Kagan, 2020; Lim et al., 2022). To activate TLR3, a minimum dsRNA duplex of 90bp is required, but the avidity of TRIF to the receptor increases as more receptors cluster together, which results in an increased signal strength on longer dsRNA (Leonard et al., 2008; Jelinek et al., 2011; Luo et al., 2012). However, the dsRNAs that are detected by TLR3 can be much shorter than dsRNA detected by MDA5 and NRLP1 (Table 1). Furthermore, TLR3 recognition is not dependent on the end structure of the dsRNAs, which makes it possible to detect dsRNA duplexes in the same range as RIG-I, but without the requirement for a tri- or diphosphate on the 5′end. However, incorporating nucleoside modifications like m6-adenosine or s2-uridine lead to reduced TLR3 activation (Karikó et al., 2005).

Protein kinase R (PKR) is a protein kinase whose enzymatic activity is dependent on dsRNA binding. Upon binding a dsRNA, two PKR proteins are stabilized by autophosphorylation and become active (Dey et al., 2005) (Figure 2B). The active kinase can then phosphorylate the eIF2 transcription initiation factor, which subsequently shuts down protein synthesis (Balachandran et al., 2000). Moreover, PKR seems to play a role in modulating other RNA sensors through its involvement in the formation of stress granules (SG) (Onomoto et al., 2012) (Figure 2B). In several studies recruitment of RIG-I, MDA5, PKR and 2′-5′-oligoadenylate synthetase (OAS) to SG has been shown (Onomoto et al., 2012; Langereis et al., 2013; Manivannan et al., 2020; Paget et al., 2023). However, it remains unclear if this recruitment has a positive or negative effect on immune signaling (Yoo et al., 2014; Paget et al., 2023). Another cell condensate that recruits PKR is dsRNA-induced foci (dRIFs) (Corbet et al., 2022; Zappa et al., 2022) (Figure 2B). These dRIFs form predominantly before protein synthesis is suppressed and contain proteins that are also involved in dsRNA sensing (NLRP1) or regulating the immune response (DHX9, ADAR1) (Corbet et al., 2022; Ren et al., 2023; Levanon et al., 2024). As with SG, the data available for dRIFs condensates do not yet provide a clear conclusion on whether these dRIFs are amplifying or controlling the dsRNA sensing response (Corbet et al., 2022; Zappa et al., 2022). In any case, to activate PKR a minimum length of 30 – 33bp is required and the maximum level of activation positively correlates with the length of the dsRNA (Husain et al., 2012). Furthermore, it was shown that ssRNAs that form a short stem-loop were also detected by PKR, even though the activation was also dependent on the presence of 5′triphosphates (Nallagatla et al., 2007). This highlights the possibility that PKRs also detect the secondary structure of a correctly transcribed RNA product. In contrast, the presence of pseudouridine (Ψ) in the RNA has been shown to reduce PKR activation and boost translation (Anderson et al., 2010).

2′–5′ Oligoadenylate synthetases (OAS) are, like PKR, enzymes whose catalytic activity is dependent on dsRNA. In humans, the family is comprised of OAS1 – OAS3 and OASL, with OAS1 – OAS3 possessing enzymatic activity while OASL does not (Rebouillat and Hovanessian, 1999). Active OASes fuse ATP molecules together by linking the 2′ and 5′ positions of the ribose rings (2′p5′a) (Hovanessian and Justesen, 2007; Kristiansen et al., 2011). From these 2′5′- linked ATPs the OASes catalyze the formation of even longer oligomers that lead to activation of Ribonuclease L (RNase L), which degrades ssRNA (Han et al., 2014; Hornung et al., 2014) (Figure 2B). Even though OAS1 – 3 all harbor enzymatic activity, OAS3 is the prime activator of RNase L, leaving the main function and physiological role of OAS1 and OAS2 open (Ibsen et al., 2014; Sarkar et al., 2023). The minimum duplex lengths that are required for activation vary between ∼20bp for OAS1 to ∼35bp for OAS2 and ∼50bp for OAS3, even though the mechanism of dsRNA binding and activation is still unresolved (Donovan et al., 2015; Koul et al., 2020; Wang et al., 2020). OASL, although also capable of binding dsRNA without having an enzymatic activity, is thought to influence the immune response through other pathways, like activation of RIG-I signaling, but mechanistic insights remain unclear (Rex et al., 2023). Even though the binding mechanism is unknown, it was shown that RNA with modified nucleotides like pseudouridine, m6-adenosine and s2-uridine has a reduced capability to activate OASes and is resistant to degradation by RNase L (Anderson et al., 2011).

### RNA:DNA hybrids

During IVT the newly synthesized RNA strand can displace the nontemplate strand from the DNA duplex and anneal to the template strand of the DNA template, thus forming stable RNA:DNA hybrids. The amount of these hybrid byproducts depends on the sequence of the transcribed DNA template. IVT of purine-rich sequences or DNA templates containing multiple GAA repeats using T7 RNA polymerase has been demonstrated to yield significant amounts of RNA:DNA hybrids (Daniels and Lieber, 1995; Grabczyk et al., 2007). These generally underappreciated IVT contaminants can be detected by immunoblotting using established anti-RNA:DNA hybrid antibodies (Boguslawski et al., 1986; Phillips et al., 2013). There is increasing evidence that the PRRs cGAS (Mankan et al., 2014), TLR9 (Rigby et al., 2014) and NLRP3 (Kailasan Vanaja et al., 2014) can detect RNA:DNA hybrids leading to the activation of innate immune signaling pathways and the induction of cytokine, chemokine and type I interferon expression. This strongly suggests that RNA:DNA hybrid contaminants are also sensed by innate immune receptors and thus, contribute to the immunogenicity of IVT mRNA. However, studies analyzing this possibility are currently lacking. Nevertheless, effective removal of contaminating RNA:DNA hybrids from clinical grade IVT mRNA should be considered if immune activation is not desirable in a therapeutic setting.

Removal of the dsDNA template from IVT reaction mixes is usually achieved by digestion with DNase I directly after the completion of the IVT process. DNase I hydrolyzes ssDNA and dsDNA as well as the DNA strand of RNA:DNA hybrids. The specific activity of DNase I for RNA:DNA hybrids, however, is at least 100-fold below that for dsDNA (Sutton et al., 1997). Compared to the dsDNA template, DNase I therefore removes the RNA:DNA hybrid contaminants very inefficiently from IVT reactions. Residual RNA:DNA hybrids may thus be co-isolated together with the single-stranded mRNA from the IVT reaction mix by established methods like precipitation with lithium chloride or alcohol/sodium acetate, or by using nucleic acid binding silica material. In contrast to dsRNA, RNA:DNA hybrids are also not removed by cellulose-based chromatography (Baiersdörfer et al., 2019). Therefore, there is a need to establish methods for efficient elimination of RNA:DNA hybrids from IVT mRNA like HPLC (high-performance liquid chromatography) purification or optimized conditions for enzymatic digestion.

## IVT reaction contaminants/impurities of non-nucleotide nature and their biological effects

In addition to the intrinsic biochemical nucleotide-based byproducts produced during IVT, other types of exogenous contaminants or impurities that may be present in the final product could have a biological impact. Examples of these potential contaminants are residual solvents, enzymes such as RNases, endotoxins and metal ions. The source of these contaminants may be from the starting materials or the associated procedures such as template production, IVT reaction, purification, filtration, formulation, and packaging. Generally, GMP drug production relies on strict quality control of both the starting materials and the final drug substance. Special attention should be given to all possible extractables/leachables for all materials with liquid product contact. Since these contaminants, similar to nucleotide-based byproducts, may lead to significant biological effects, there is a necessity for strict mRNA production and quality assessments covering all types of potential contaminants and impurities not only for clinical studies but also for any research or preclinical studies.

### Ribonucleases (RNases)

RNase-free conditions are an absolute necessity for successful RNA production independent of the scale or downstream application. RNases can have diverse intrinsic cellular roles in relation to tRNA and rRNA maturation, quality control, mRNA decay and RNA metabolism in bacteria and eukaryotic cells (reviewed in Cerritelli and Crouch, 2009; Cook et al., 2013; Bechhofer and Deutscher, 2019). However, contamination with endo- or exoribonucleases can be a major cause of IVT mRNA degradation, impacting its integrity and compromising its drug activity. For example, RNA interferases are endonucleases belonging to the bacterial toxin-antitoxin (TA) systems and have a major role in degrading mRNA and inhibiting mRNA translation, thus preventing bacterial growth (Cook et al., 2013). Culviner et al. used template switching libraries in combination with RNA-seq and found that a number of *E.coli* endoribonucleases cleave mRNA at short low-complexity cleavage motifs exemplified by ACA for MazF and UAC for ChpB mRNA interferases (Culviner et al., 2021). Moreover, the authors found that three interferases from the ReIE family cleave before a G, showing minimal specificity (Culviner et al., 2021). In addition to interferases, a number of other RNase subtypes exist that can contaminate mRNA production (reviewed in Bechhofer and Deutscher, 2019): (1) endonucleases such as RNase III, RNase HII and RNase P; (2) 5′exonucleases such as RNase J1 and J2; and (3) 3′endonucleases such as PNPase, RNase PH and RNase R. In all cases using contaminant-free starting materials, RNase-free consumables, reducing handling and production time, proper mRNA storage conditions, and in the future, avoiding usage of templates produced in *E. coli* (Table 2), may significantly contribute to the prevention of RNase contamination in the IVT reaction or the formulated drug.

**TABLE 2:** Upstream and downstream solutions to reduce IVT reaction byproducts and impurities.

Impurities	Upstream precautions	Downstream purifications
RNases	Sterile conditions, well-defined RNase-free starting materials, RNase-free consumables, reducing handling and production time, proper mRNA storage conditions, avoiding usage of templates produced in *E. coli,* usage of RNase inhibitors	Chromatography, filtration
Endotoxins	Strictly endotoxin-free starting materials and consumables, avoiding usage of templates produced in *E. coli*	Chromatography, filtration
Metal ions	Well-defined starting materials, attention to potential leachables including containers, closures, storage bags	ND
dsRNA	Improved IVT conditions (pH, temp, adding auxiliary reagents), mutated RNA Pol II, promoters, optimized DNA template	Cellulose chromatography, oligo(dT) bead purification, HPLC
Abortive RNAs	Pyrophosphorolysis, mutated RNA Pol II, transcription start site/sequence optimized DNA template	HPLC
Degraded RNA	RNase inhibitors, lower IVT reaction temperature	HPLC
RNA:DNA hybrids	DNAse I treatment	HPLC

Abbreviations: HPLC, high-performance liquid chromatography; IVT, *in vitro* transcription; mRNA, messenger RNA.

### Endotoxin

Endotoxin contamination of mRNA-based therapeutics might originate from linearized plasmids produced in *E. coli* used as templates for IVT reactions, or from any of the raw materials including water. Endotoxins originate from the outer membrane of Gram-negative bacteria. They are composed of lipopolysaccharide (LPS), consisting of an acylated backbone (lipid A) responsible for the induction of a proinflammatory immune response, a chain of repeated sugar units (O-antigen) and an oligosaccharide core (O'Neill et al., 2013; Cavaillon, 2018; Sondhi et al., 2024). If present, LPS contaminants can interact with LPS-binding protein (LBP), Myeloid differentiation protein 2 (MDA2), CD14 and Toll-Like Receptor 4 (TLR4), thereby leading to signal transduction through the TRIF and MyD88 pathways, resulting in the secretion of proinflammatory cytokines such as IL-6, TNF-α, and IL-1 (O'Neill et al., 2013; Sondhi et al., 2024). In addition, LPS can interact with caspase-4 in mice or caspase-4/5 in humans, leading to inflammasome activation and IL-1α and IL-1β release (Kayagaki et al., 2011; Viganò et al., 2015). Endotoxin is extremely potent, where even amounts as low as 0.1–0.5 ng/kg can lead to cytokine release, and dose-dependent side effects such as fever, chills, nausea, hypotension, tissue damage, sepsis, and death might occur (Elin et al., 1981; Sondhi et al., 2024). Thus, the upper limits and acceptance criteria as well as analytical methods for measuring endotoxin are strictly defined per USP chapter <85> Bacterial Endotoxins Test 3 (BET) based on dose, application route and type (Dawson, 2017). In addition to endotoxin, bioburden and sterility are part of the critical quality attributes (CQAs) ensuring the safety of mRNA products with regard to microbiological contamination (BioPhorum Operations Group Ltd, 2023).

### Metal ions

The potential impact of metal ions on mRNA-based drugs is not fully understood. At high concentrations, heavy metal ions can bind with varying affinities to specific motifs in RNA. They can modify its secondary or tertiary structure and influence folding, potentially negatively affecting RNA stability (Pyle, 2002; Draper, 2004). Searching for specific RNA-ion interactions, Ciesiolka et al. performed Zn^2+^ affinity selection of a large RNA pool and detected GC cluster, the augmented GC cluster (two adjacent helical GC base pairs) and E element (consisting of AUG and AAC triads) as motifs, which together with conserved flanking G:C and C:G pairs, bind Zn^2+^ (Ciesiolka and Yarus, 1996). Hofmann et al. proposed that small, asymmetric, purine-rich loops containing G-A interactions may represent binding sites for divalent metal cations (Hofmann et al., 1997). For example, they used *in vitro* selection and amplification with a polymer matrix that contains Ni^2+^-nitrilo-tri-acetic-acid and showed binding of Ni^2+^ to such specific conserved RNA motifs present in their screen (Hofmann et al., 1997). Furukawa et al. discovered a riboswitch czc motif RNA that forms specific binding pockets which interacted strongly with Co^2+^ and Ni^2+^, and weakly with Mn^2+^ (Furukawa et al., 2015). Interestingly, in-line probing analysis of the same sequence resulted in spontaneous RNA degradation when La^3+^, Os^3+^, Sn^2+^, Hg^2+^ and Pb^2+^ were tested. Sequence-specific Pb^2+^-induced hydrolysis of RNA was already described in model RNA oligomers and further noted as potentially useful for interpreting the cleavage of large mRNAs (Ciesiołka et al., 1998). Kiliszek et al. recently found that a UGG motif can bind up to two metal ions (Ba^2+^, Cs^+^ and Sr^+^) (Kiliszek et al., 2022). The presence of Ba^2+^ was associated with two G · U and G · G wobble noncanonical pairs formed by a UGG/UGG motif that significantly impacted the structure of the RNA. While in the majority of cases the ion-binding motifs in RNA were examined in the context of ribozymes and riboswitches, where ions play important roles in their activity, the potential impact on the folding and stability of mRNA drugs by excess ion contaminants binding to structural motifs is still unknown. In addition, the dual role of some cations (e.g., Mg^2+^) in: 1) intrinsic in-line mRNA hydrolysis and 2) increasing the activity of certain RNases, may promote RNA instability (AbouHaidar and Ivanov, 1999; Sun et al., 2005). Therefore, regarding undesirable metal ion contaminants, special attention should be given to potential leachables including containers, closures, storage bags, etc. (Table 2) early in process development as well as in the research laboratory for small scale mRNA production.

## Overcoming or depleting nucleotide-based byproducts and contaminants/impurities from IVT reaction

### Strategies to prevent the formation of abortive byproducts

To reduce the generation of abortive byproducts during IVT reactions several parts of the reaction can be altered, including using mutant forms of the T7 RNA polymerase, changing the T7 promoter sequence, optimizing the sequence of the transcription start site or adding pyrophosphatase (Table 2).

#### Mutating the T7 RNA polymerase

Using *E. coli* expressing a combination of a mutant version of the T7 RNA polymerase (I810S mutant) and the lacZ enzyme with an unfavorable transcription start site, Guillerez et al. were able to abolish productive transcription of the T7 RNA polymerase and ‘trap’ the enzyme in abortive cycling (Guillerez et al., 2005). After hydroxylamine-mediated mutagenesis of the plasmid encoding T7 RNA polymerase, they screened for restoration of productive transcription based on the expression of lacZ and identified the P266L mutation as a potent way of reducing the generation of abortive products while increasing synthesis rate and maximum yield. Guillerez et al. proposed weakened promoter interactions to be causal for the reduced amount of abortive products (Guillerez et al., 2005).

Later it was found that the P266L mutant leads to a reduction of very short abortive byproducts (4–7 nt in length) since it is transitioning at longer length into the elongation phase (onset of transition is 2 nt later than for the WT polymerase) (Ramírez-Tapia and Martin, 2012; Tang et al., 2014). As described above, the newly synthesized RNA in form of a growing RNA:DNA hybrid ‘pushes’ against the promoter-bound (N-terminal) region of the RNA polymerase and the promoter-bound region due to the promoter binding affinity *vice versa* “pushes” against the RNA:DNA hybrid, which “destabilizes” the RNA:DNA hybrid and eventually leads to the release of abortive byproducts. The P266L mutation is thought to decrease the rigidity of the rotation of the N-terminal domain, allowing a longer and thus more stable RNA:DNA hybrid pushing against the promoter-bound region and thereby decreasing the probability of an abortive release (Ramírez-Tapia and Martin, 2012; Tang et al., 2014).

Although the P266L mutant drastically reduces the amount of abortive byproducts 4 to 7 nt in length, it should be noted that the mutant generates abortive byproducts of longer length (9–11 nt) which might show different effects in transfected cells than shorter byproducts. The beneficial effect of the later transition into the elongation complex, which significantly reduces very short abortive byproducts, comes at the expense of a less favorably positioned 5’ end of the RNA which needs to find its way into the RNA-exit channel of the RNA polymerase (the previously promoter-bound region of the enzyme contributes to the formation of an RNA-exit channel after its rotation). As a result, RNA synthesis is (slightly) more likely to stop after 11–13 nt.

Using the P266L mutant in combination with a mutated promoter known to weaken upstream promoter interactions (cytosine instead of adenosine (A-15C) in the φ9 promoter), all species of abortive byproducts could be reduced–shorter ones due to the use of the P266L mutant, longer ones due to the φ9(A-15C) promoter–as it allows the P266L mutant to undergo the transition from initiation to elongation ‘already’ at 9 nt, similar to the WT enzyme. This results in a more efficient release from the promoter and fewer long abortive byproducts (Tang et al., 2014). Consequently, the combination of the P266L mutant enzyme with the φ9(A-15C) promoter yields a significantly higher proportion of productive vs. abortive synthesis compared to the WT enzyme. These results suggest that this combination could represent a valuable tool for large-scale mRNA production.

#### Pyrophosphorolysis

An increasing amount of diphosphate (PPi) occurring through the incorporation of NTPs can shift productively cycling *E. coli* RNA polymerase complexes to abortive cycling, leading to an increase of primarily short 3 to 5 nt byproducts (Plaskon et al., 2022). Thus, when including pyrophosphatase during the IVT reaction (as reported first by Sampson and Uhlenbeck, 1988) using T7 RNA polymerase, it not only increases the general yield of the reaction (Cunningham and Ofengand, 1990), but might also lower the number of abortive byproducts.

#### Nicks, gaps and truncated promoters

Abortive byproducts can arise due to the instability of the initially generated RNA:DNA hybrid that needs to ‘loosen’ the promoter-bound subdomain of the enzyme by causing the drastic 220° rotation of the enzyme into the elongation conformation. Thus, it was hypothesized that weakening the promoter interactions of the T7 RNA polymerase might facilitate the promoter release, thereby stabilizing the RNA:DNA hybrid and finally leading to less abortive byproducts. Truncated promoters were shown to yield successful and normal levels of transcription, but also comparable levels of abortive byproducts, when the DNA in the nontemplate strand was removed at position −17 to −14 and −3 to +6 (Milligan et al., 1987). Similarly, a mismatch at position −10 or a truncation of the nontemplate strand upstream of position −14 were described to result in only slight changes in the rate of abortive byproducts formation despite having an order of magnitude lower affinity (Km) (Martin et al., 1988).

In a study by Jiang et al. a variety of promotor topologies were tested: non-complementary nontemplate strands in position −4 to +5, insertion of six non-complementary nucleotides in the nontemplate strand (between −5 and −4), and deletion downstream of −5 (Jiang et al., 2001). Also nicks in the template strand between −5 and −4 or gapped strands (deletion of −3 and −4) were tested. Moreover, they introduced 6 nt insertions (between −5 and −4) in the template strand whereby the nontemplate strand was either fully complementary (including the insertion), complementary except for the insertion (non-complementary insertion), or was lacking an insertion or was non-complementary from position −5 to +5. For those templates that resulted in the production of a relevant amount of run-off products, only the nicked DNA template strand at position −5 not only reduced the formation of abortive products significantly but also the overall yield of the reaction (Jiang et al., 2001). Thus, reducing the amount of abortive byproducts using nicked, gapped or truncated promoters seems to be unsuitable for a large-scale production of RNA therapeutics.

#### Transcription start site/sequence optimization

Incorporation of uridine monophosphate (UMP) in the first eight transcribed nucleotides was shown to result in the highest probability of abortive initiation whereas guanosine monophosphate (GMP) incorporation had the least probability for generating abortive byproducts (Martin et al., 1988). Consistent with this finding, Imburgio et al. noted that all natural T7 promoters/transcription start sites do not encode for an UMP incorporation until position +6 (Imburgio et al., 2000). Incorporation of UMP at position +3, however, increased the number of abortive products (Imburgio et al., 2000).

### Strategies to prevent the formation of dsRNA and 3’ nucleotide addition

#### Optimizing IVT reaction conditions

To avoid the emergence of dsRNA byproducts during IVT, many reaction parameters have been tested, including the concentration of ions, chaotropic agents, nucleotides and temperature (Table 2). Lowering the ion concentrations of Mg^2+^ from 30 mM to 5 mM led to a reduced amount of dsRNA (Mu et al., 2018). Even though the precise mechanism is unclear, one hypothesis is that a high Mg^2+^ concentration stabilizes the EC which keeps the T7 RNA polymerase transcriptionally active thereby generating elevated levels of dsRNA (Mu and Hur, 2021). Another approach to prevent the production of dsRNA is to add chaotropic agents like urea or formamide to the IVT to weaken undesired binding of the poly(A) tail to the product and thereby prevent backwards transcription. With this strategy a reduction of the dsRNA content of about 60%–70% could be achieved (Piao et al., 2022). As an alternative to chaotropic agents to prevent the backfolding of the product, the reaction temperature during IVT can be increased. Interestingly, studies with thermostable T7 RNA polymerase showed that temperatures of up to 50°C were effective to reduce dsRNA by 3′ extension, but failed to reduce dsRNA formation by strand switching of the T7 RNA polymerase (Wu et al., 2020). Apart from that, the backfolding of the RNA product can also be prevented by adding a capture DNA complementary to the 3′end of the RNA product (Gholamalipour et al., 2019). To effectively reduce 3′extension the capture DNA needs to be longer than 14 nucleotides and has to be present in excess compared to the RNA product (Gholamalipour et al., 2019). Instead of preventing the backfolding of the RNA, the backward transcription can also be avoided by limiting the amount of uridine excess. This applies in particular to mRNAs with a poly(A) tail, since their backward transcription starts with a poly(U). Ziegenhals et al. were able to design a fed batch process in which the limited steady-state concentration of UTP or m1ΨTP led to reduced dsRNA production during IVT (Ziegenhals et al., 2023). Furthermore, they found that di-nucleotide caps had an improved dsRNA profile compared to tri-nucleotide caps. In addition to the excess of certain nucleotides, the nucleoside modification also plays a role. The use of Ψ, m1Ψ and m5C nucleosides led to lower dsRNA production (Baiersdörfer et al., 2019; Mu and Hur, 2021).

#### Mutating or switching RNA polymerase

Next to optimizing reaction conditions, a viable option is also to mutate the T7 RNA polymerase or change the polymerase altogether. The carboxy-terminal domain (CTD) of the T7 RNA polymerase has been shown to have an influence on 3′ homogeneity, as well as enzyme productivity. The homogeneity of the 3′end is higher the larger the amino acid that is added to the end, while the productivity decreases with increasing size (Gardner et al., 1997; Dousis et al., 2023). Since the EC has been proposed as the driving force behind the dsRNA byproducts, stabilizing the IC might be favorable to reduce dsRNA. The mutation G47A has been identified to reduce dsRNA production without loss of yield and computational analysis suggests that it favors the IC over the EC, even though the equilibrium studies are still ongoing (Dousis et al., 2023). While T7 is by far the best optimized and characterized RNA polymerase, several other phage RNA polymerases have surfaced in recent years. All of them emerged from the subfamily of the short-tailed phage *Autographiviridae*, which was also the origin of the T7 RNA polymerase (Lu et al., 2019). The characterized polymerases include T3, SP6, Syn5 and KP34 polymerases (Butler and Chamberlin, 1982; Zhu et al., 2013; Lu et al., 2019). Even though claims of higher yield and lower byproducts have been announced for most of them, it remains to be shown that these polymerases are valid competitors for T7 under high yield conditions in an industrial setting (Zhu et al., 2014; Lu et al., 2019). The latest candidate that emerged is the VSW-3 polymerase from the psychrophilic bacteriophage VSW-3 (Xia et al., 2022). In laboratory scale under VSW-3-optimized conditions VSW-3 polymerase shows a higher 3′ homogeneity and comparable yield to T7, while it has a lower optimal reaction temperature of 25°C, which would reduce RNA degradation during the IVT (Wang et al., 2022). Interestingly, the lower temperature leads in this study to lower dsRNA byproducts, contrary to the other study that depleted dsRNA with a thermostable T7 RNA polymerase and higher temperature (Wu et al., 2020). These results leave open the question of the fundamental mechanisms driving the formation of dsRNA byproducts by this new polymerase. Additionally, it should be noted that since the mechanism of 3′extension is dependent on the concentration of the run-off product, lower yield reaction will always lead to less dsRNA byproducts, making it difficult to draw conclusions about high yield conditions from these studies (Gholamalipour et al., 2018; Mu et al., 2018).

#### Optimization of the DNA template

Lastly, not only the IVT itself and its components and reaction conditions have an influence on dsRNA formation, but also the DNA template from which the RNA is produced. Especially the preparation and the purity of the linearized template has an impact on dsRNA. It was demonstrated with monolithic columns with butyl chains, which are normally used to fractionate nucleic acids from proteins by hydrophobic interactions, that the purity of the linearized plasmid influences dsRNA content (Martínez et al., 2023). Moreover, it has long been known that the DNA template end structure has an influence on byproducts (Schenborn and Mierendorf, 1985). This was confirmed recently when Mu et al. found that a 3′ overhang at the end of the template led to significantly more dsRNA and a 5′ overhang can reduce the dsRNA content (Mu et al., 2018). In another approach, a DNA template which is single-stranded, except in the T7 promoter region, yielded significantly less dsRNA (Mu et al., 2018). High salt concentrations have been known to reduce protein-nucleic acid interactions. In the case of the T7 RNA polymerase this leads to weaker binding of the T7 RNA polymerase to the RNA product, which prevents undesired 3′extension but also weakens the binding to the T7 promoter and results in decreased yield (Cavac et al., 2021). To overcome the loss in yield, the T7 RNA polymerase needs to be forced into close proximity to the T7 promoter or have an improved binding affinity to the T7 promoter. The latter can be achieved by a small manipulation of the DNA template (Malagoda Pathiranage et al., 2023). By introducing a nick to the −4 position into the DNA template the binding affinity from the T7 to the promoter is enhanced. This has been explained by the T7 RNA polymerase using parts of the available energy from the T7 RNA polymerase–promoter complex to drive melting of the DNA downstream of the promoter (Bandwar and Patel, 2002; Martin et al., 2005). The nicked template in combination with high salt concentration has been shown as a promising approach to deplete 3′ extensions without disrupting yields (Malagoda Pathiranage and Martin, 2023).

### Depleting byproducts and impurities by purification

Another option to remove byproducts from IVT is purification and filtration following IVT, where the full-length mRNA is separated from all diverse types of impurities and contaminants (Table 2). There are several methods to ensure purified, high-quality mRNA, and they are proven to remove both process related impurities (plasmid DNA fragments, enzymes, unused NTPs or cap analogs, and additive ingredients) and product-related impurities (truncated ssRNA, DNA-RNA hybrids and dsRNA). Since mRNA can be sensitive and degrades easily under certain conditions, rapid and selective purification is required. During purification procedures, the elimination of product-related impurities imposes specific challenges since those species share similar physicochemical properties with the final mRNA product.

In the laboratory under small scale production conditions, mRNA is typically precipitated using alcohol and sodium or ammonium salts or by using lithium chloride alone (Walker and Lorsch, 2013). Large-scale mRNA production may however integrate diverse non-chromatography or chromatography-based mRNA purification methods (as previously and extensively reviewed in Baronti et al., 2018; Zhang et al., 2023), and these can be combined as well. Among the combined methods, the “sandwich” method is widely used, where a chromatography-based purification is flanked both before and after by tangential flow filtration (TFF) steps. During TFF, through tangential continuous flow, the mRNA is separated from impurities in the IVT mixture by filtration through a membrane, and subsequently the mRNA solution can also be concentrated (Table 2). In the chromatography step, molecules are separated based on their physicochemical properties, e.g., charge, size and hydrodynamic radius, affinity to specific ligands or differences in hydrophobicity (Karikó et al., 2011; Linares-Fernández et al., 2021; Szabó et al., 2022). Affinity chromatography in the case of mRNA purification utilizes a resin functionalized with oligo-deoxythymidylic acid (oligo-dT) to capture mRNA by forming bonds with its poly(A) tail. There are diverse ways to implement this purification strategy, e.g., by using magnetic oligo (dT) beads (Jacobsen et al., 2004; Green and Sambrook, 2019; Korenč et al., 2021). Despite these purification methods, the solution still may contain dsRNA, and thus consequently may need to be combined with further downstream purifications. For example, high-performance liquid chromatography (HPLC) (Karikó et al., 2011) and cellulose-based columns (Baiersdörfer et al., 2019) have been proven to significantly mitigate dsRNA contamination (Table 2).

## Conclusions and future perspectives

*In vitro* transcription (IVT) is the fundamental process used to generate synthetic mRNA transcripts at scale for therapeutics. All mRNA-based vaccines and drugs currently on the market or in clinical trials rely on the IVT reaction to be produced. Despite the advancements made to the IVT reaction, in the majority of cases it still produces byproducts and there are persistent challenges in scalability and mRNA purification. In the future, IVT might be replaced by new technologies. One such strategy is cell-based mRNA manufacturing, which is currently being exploited in publicly funded and commercial projects. While these efforts aim to develop cell-based factory platforms for large-scale and cost-effective manufacturing of mRNA therapeutics, this technology still requires its own process optimizations. Furthermore, some potential limitations of such an approach might be the inability to generate modified mRNA (such as N1-methylpseudouridine- or pseudouridine-modified mRNA) and the possibility of uncontrolled cell-based modifications and non-optimal cap structures. These issues could negatively impact the biological activity of mRNA in human cells. Another future strategy would aim at synthesizing long mRNAs chemically; however, new advancements are also required in that field since it is currently limited to sizes of about 100 nucleotides. In the meantime, the enzymatic IVT reaction continues to be the standard, most reliable and cost-effective mRNA manufacturing method available as it has been for more than 40 years. As discussed, numerous optimizations, including improvements to the RNA polymerase, starting materials and reaction conditions, as well as refinements to downstream purification methods, have led to an increased yield of high-grade, full-length mRNA and a decrease in byproducts, contaminants, and impurities. Consequently, this has greatly improved both the efficacy and safety of mRNA therapeutics. Further engineering of the RNA polymerase and IVT reaction conditions, as well as tailoring mRNA purification to diverse medical applications is ongoing. For example, both the drug substance and byproducts can induce innate immune activation that might also impact adaptive immunity, and this may be beneficial for certain applications such as vaccines or fully undesirable for others such as RNA protein replacement therapies. These differences may necessitate specific manufacturing requirements for diverse medical applications. In addition, complete characterization of certain byproducts and impurities depends strongly on sensitive and reliable analytical methods that are also constantly under development. Here we have discussed the biological impact of some byproducts, for example, dsRNA, which in high quantities leads to cytokine and chemokine release and thus potential safety issues. However, a better understanding of the types, subclasses and quantities of all byproducts and impurities as well as defining their biological impact in different cell types, including in diverse application routes would be highly beneficial for characterizing mRNA drug activity and safety. For example, it is still challenging to detect and quantify dsRNA byproducts smaller than 50bp and therefore difficult to understand their biological effects. Moreover, the biological impact of abortive RNA byproducts originating from the IVT reaction also remains elusive in the context of RNA therapeutics. Thus, further research of the biological effects of IVT reaction byproducts and impurities, together with improved analytical methods will help in future tailoring of mRNA drug production for specific medical applications.

## Abbreviations

dsRNA, double-stranded RNA; LPS, lipopolysaccharide; mRNA, messenger RNA; NTP, nucleoside-triphosphates; ssRNA, single-stranded RNA.
